# Efficacy of Manual Therapy *versus* Conventional Physical Therapy in Chronic Low Back Pain Due to Lumbar Spondylosis. A Pilot Study

**DOI:** 10.3390/medsci3030055

**Published:** 2015-06-26

**Authors:** Arti Sharma, Khalid Alahmari, Irshad Ahmed

**Affiliations:** Department of Medical Rehabilitation Sciences, College of Applied Medical Science, King Khalid University, Abha 61321, Saudi Arabia; E-Mails: artiphysio@gmail.com (A.S.); kahmarie@kku.edu.sa (K.A.)

**Keywords:** maitland, mobilization, exercise, traction, low back pain

## Abstract

Objectives: The objective of this work was to compare the efficacy of Maitland mobilization and conventional physical therapy on pain response, range of motion (ROM) and functional ability in patients with chronic low back pain due to lumbar spondylosis. Methods: A total sample of 30 subjects (40–70 years of age) with complaints of slow insidious onset of low back pain (LBP), with or without radiation not less than three months duration and decrease ROM were randomly assigned to: group-I, Maitland mobilization and lumbar stabilization exercises; group-II conventional physical therapy (traction, strengthening, stretching exercises.) and outcomes were assessed for dependent variables. Results: There is statically a significant difference between pre and post measurement readings with time (*p* = 0.00) and between groups (*p* < 0.05) with respect to pain and function, but, with respect to ROM readings, showed statistical significance with time (*p* = 0.00) and no significance between groups (*p* > 0.05), indicating manual therapy group-I is improving faster and better than conventional physical therapy group-II. Conclusion: Our results showed that manual therapy interventions are more effective in managing low back pain, and function and range of motion of the lumbar spine than conventional physical therapy treatment.

## 1. Introduction

Chronic low back pain due to lumbar spondylosis, is defined as aching low back with or without radiation to lower limbs not less than three months in duration, with confirmed signs of degeneration in lumbar spine on X-Ray [1]. The main feature is pain in lumbar region, often accompanied by restriction in range of motion (ROM) and functional limitation. Risk factors include age, heredity, impact of activity, and occupation. Low back pain and its related disabilities cause an important socioeconomic burden to society [2] and is the most common cause of absence from work [3]. Manipulations, mobilizations, and exercise are favored over traditional care in reducing chronic low back pain at both short-term and long-term follow-ups. A systematic review by Rothschild [4] studied whether conservative treatments (e.g., manual therapies, physical medicine methods, medication, and patient education) relieved pain or improved function/disability, patient satisfaction, and global perceived effect in adults with chronic low back pain. Results of this review revealed that exercise combined with mobilization/manipulation demonstrated either intermediate or long-term benefits. Critical review of literature and randomized controlled trials by concluded manipulation, mobilization, or exercise are beneficial in patients suffering from chronic low back pain when applied as single-modal treatment approaches [5,6,7]. Different forms and techniques in manual therapy exist including both manipulation and mobilization and all use hands as a common feature during therapy [8]. Studies have shown that manual therapy techniques provide effective relief for chronic low back pain [5,7,9,10]. These techniques include manipulation (*i.e.*, a high velocity thrust directed at the spinal joints) and mobilization techniques that do not involve a high velocity thrust. Very few studies have looked into the efficacy of manual therapy on chronic low back pain [10,11,12], and some have found that lumbar mobilization, using the Maitland technique, relieves pain and normalizes function [10,11]. High-quality evidence suggests greater short-term pain relief from manual therapy than exercise alone, but no long-term differences were found for chronic low back pain [11]. Maitland mobilization is one of the most common manual therapy approaches used by physical therapists [13]. Maitland mobilization is a passive oscillatory technique, applied over the hypo-mobile vertebra level, and the methods are considered valid [14]. Further, no work has been done to observe the effects of Maitland mobilization and exercises over conventional treatment, to date, in the management of chronic low back pain. This has provided the authors with focus in this work. 

## 2. Methodology

Sampling size: A total number of 30 subjects of both sexes age between 40–70 years diagnosed with chronic low back pain due to lumbar spondylosis participated in this study. 

### 2.1. Inclusion Criteria

1. Chronic low back pain with or without radiation not less than three months duration.
2. Age groups—40 to 70 years.
3. Sex—male and female.
4. History of slow, insidious onset of pain.
5. Physical examination.
  - Limited ROMs of lumbar spine like extension, flexion or side flexion.
  - Extension may be more limited than other movements.
  - Pain during extension like standing and relieved by flexion like sitting.
  - Pins and needles sensations
    (two out of over four should be there).
6. Nature of pain-aching pain, feeling of heaviness in legs, intermittent burning or numbness.
7. No neurological deficits.
8. Diagnosis confirmed by X-Ray showing signs of degeneration.

### 2.2. Exclusion Criteria

1. Prolapse with neurologic signs and symptoms requiring surgery.
2. Pregnancy.
3. Spondylolisthesis.
4. Spondylolysis.
5. Mechanical strain.
6. Degenerative listhesis.
7. Fractures.
8. Suspicion of malignancy.
9. Osteoporosis.
10. Previous back surgery.
11. Known rheumatic, neurologic, or mental diseases.
12. Absence of pain aggravation on active, functional movement tests (*i.e.*, indicating nonorganic symptoms).
13. Other red flags (contra-indications) to manual therapy.

### 2.3. Variables: Independent Variables: Independent Variables of This Study Were

1. Manual therapy.
2. Stretching exercises and traction.
3. Lumbar Stabilization exercises.

### 2.4. Dependent Variables of This Study Were

1. Spinal range of motion measurement by the modified Schober’s test—Extension, flexion, and side flexion.
2. Functional disability measurement using the Oswestry Low Back Pain Disability Questionnaire.
3. Average VAS (Visual Analogue Scale) for pain at rest and activity.

### 2.5. Materials and Methods

A prospective repeated-measures design was used to determine the efficacy of two interventions during a four-week program. Each measurement was taken two times: at baseline level (pretest) and after the last session of intervention, *i.e.*, after four weeks of therapy (post-test).

This study was approved by an ethical and research committee. Sample size was calculated and determined at 30 participants (15 in each group) to find a between-group difference in pain (visual analog scale (VAS), 11-pointscale (0–10)), with the power established at 80%, and significance level at 0.05. Participants were recruited from the Department of Medical Rehabilitation Sciences, King Khalid University. Physicians and physical therapists which were posted in the out-patient department were requested to refer patients complaining of chronic low back pain to the place of study. Ninety-two participants (*m* = 41; *f* = 51) were examined and were included in the study if they satisfied the inclusion criteria. All subjects were tested using pretest measurements, which included calculation of spinal range of motion measurement, as per the modified Schober’s test, functional disability measurement, using the Oswestry Low Back Pain Disability Questionnaire, and average VAS at rest and while active. Forty-five participants (*m* = 18; *f* = 27) were excluded and 17 participants (*m* = 6; *f* = 13) who expressed their inability to attend therapy regularly were excluded. Participants recruited were from geographically different units within the state and were randomized into two different groups, designated as groups I and II. Thirty participants (14 females and 16 males) with a mean age of 57.33 years participated and completed the study.

Participants attended a preliminary screening session. After explaining the needs and purpose of the study, a duly signed consent form was obtained from each participant. Those who fulfilled inclusion criterion were then asked for their demographic details, their present and past medical history, family history, surgeries undergone, if any, and so on. Patients were then given clear instruction for measurement intervention.

## 3. Procedure

### 3.1. Group I (Maitland Mobilization and Lumbar Stabilization Exercises)

Group I: 15 participants (*f* = 6; *m* = 9) received Maitland mobilization to the lumbar spine along with lumbar stabilization exercises for a period of four weeks (five days a week, one session per day). Each session lasted almost 30 min. Treatment by the Maitland technique attempts to gauge the effectiveness of intervention by assessing segmental movement that is limited by the patient’s symptoms. Participants received Maitland mobilization, targeted at impairments identified during the physical examination. Participant was positioned in a prone position, and the treating therapist stood on the left side of the patient and positioned their left hand on patient’s back so that part of ulnar border of hand, between the pisiform and the hook of the hamate, was in contact with the spinous process of vertebra to be mobilized. A central or lateral PA oscillatory pressure was applied, by gradually moving therapist’s body weight forwards more directly over patient’s vertebral column, and oscillating movement of vertebra was obtained by a rocking movement of the upper trunk, up and down, in a vertical axis. Pressure was transmitted through the arms and shoulders over the process of hypo-mobile vertebra. The following grades were used: grades I and II, where pain occurred before motion barrier; and grades III and IV, where motion barrier was encountered before pain. This oscillatory mobilization was performed at a rate of 2–3 oscillations per second with metronome control, and a frequency of 3–4 mobilization of a joint lasting approximately 30 s each. Rest time between each mobilization was one minute.

Strengthening exercises were prescribed 2 or 3 sets of 20 to 30 repetitions for each exercise, with 30 s to 1 min of rest between each set, and without provoking pain during exercises. Strengthening and stabilizing exercises for abdominal, back, pelvic, and lower limb muscles were allowed. Each week, higher levels of exercises were given and the patient was allowed to move to the next higher level if he/she was acquainted with the previous level.

### 3.2. Group II (Traction and Lumbar Stabilization and Stretching Exercises)

Group II: 15 participants (*f* = 8; *m* = 7) received traction and lumbar stabilization and stretching exercises for a period of five sessions per week for four weeks. Lumbar stabilization exercises were the same as those given to Group I. For traction, a belt was firmly fixed around patient’s thorax and a second belt around the pelvis of the patient, facing upwards on a friction-free traction couch. The position of choice was to place the IV joint midway between flexion and extension to permit the greatest longitudinal movement. Intention of traction was not to pull vertebrae apart and produce negative intra-discal pressure. The first time, a very low weight, not greater than 13 kg, was used and this was maintained for a period not exceeding 10 min. If pain was experienced by the patient, duration and pressure of the treatment was reduced. Symptoms were relieved minimally by 13 kg of traction and, under these circumstances, strength was increased approximately 20 kg, and duration was 10 min, however, if 20 kg completely relieved symptoms (especially if they were severe), strength was reduced to an amount less than 18 kg. Traction was provided by Enraf-Nonius Eltrac-471 for 10 min.

Stretching exercises for hip flexors were given in the Thomas test position, with a hold and relax technique, 6–10 times. Piriformis and hamstring stretching was also done using a hold and relax technique, 6–10 times per session. Spinal muscle stretching was performed in a quadruped position, with a hold of position for 30 s and with repetitions (3–5 times).

### 3.3. Data Analysis 

The intra-rater reliability between the measures was calculated using the intra-class correlation coefficient (ICC), and the error between repeated measures was indicated by the 95% confidence intervals for the absolute difference between trials. The Paired Samples Test was used to observe pre- and post-intervention effect. The effects of manual therapy and conventional therapy in the case of chronic low back pain were calculated by comparing the changes in selected dependent variables using one-way ANOVA, between a factor group with two levels (manual therapy and conventional therapy), and once within the factors of (time)-pre *versus* post. Pair wise *post hoc* comparisons were done using Tukey’s HSD (Honest Significant Difference) using a significance level of 0.05. All the analyses were done by using SPSS 16 statistical software (IBN, Chicago, IL, USA).

## 4. Results and Discussion

Thirty participants (14 females and 16 males) with a mean age of 57.33 years completed the study. Table 1 lists the baseline and post-interventional scores of pain, Range of Motion (ROM) and disability for all the groups investigated in the study. Table 2 lists results of ANOVA measures. The mean difference is significant at the 0.05 level. Overall results of the study showed that both groups improved over time, compared to baseline (*p* < 0.05). Results revealed no significant differences between groups in terms of values of ROM, and analyses of variance also demonstrated no significance in groups over 4 weeks of time intervention effect between groups in improving ROM (*p* > 0.05). As depicted in Table 3, there is a significant difference between pre- and post-intervention in all seven groups (*p* < 0.05).

**Table 1:** Mean (SEM) values of outcome variables.

Group	Time	VAS (Rest)	VAS (Activity)	ODI (in score)	Extension (in cm)	Flexion (in cm)	Side Flexion Lt (in cm)	Side Flexion Rt (in cm)
I	Pre	7.6(0.300)	8.13(0.182)	69.2(2.66)	1.33(0.144)	3.5(0.176)	2.97(0.210)	3.07(0.153)
	Post	1.2(0.129)	1.6(0.187)	21.13(2.29)	3.4(0.148)	5.7(0.118)	4.47(0.165)	4.4(0.19)
II	Pre	7.06(0.27)	7.93(0.182)	70.33(1.8)	1.43(0.128)	3.37(0.12)	2.97(0.124)	3(0.155)
	Post	2.6(0.23)	3.13(0.192)	32(2.162)	3.03(0.165)	5.3(0.181)	4.23(0.161)	4.2(0.175)

VAS, Visual Analogue Scale; ODI, Oswestry Disability Index; Lt, Left; Rt, Right; cm, centimeters.

**Table 2:** ANOVA results testing for effects of intervention.

Variable	Time (*F* value)	Group (*F* value)	Time × Group (*F* value)
VAS (Rest)	1.045E3 (*p* = 0.000)	6.610 (*p* = 0.016)	19.174 (*p* = 0.000)
VAS (Activity)	2.729E3 (*p* = 0.000)	5.635 (*p* = 0.025)	68.040 (*p* = 0.000)
ODI	845.424 (*p* = 0.000)	4.554 (*p* = 0.042)	10.729 (*p* = 0.003)
Extension	330.859 (*p* = 0.000)	0.540 (*p* = 0.469)	5.359 (*p* = 0.082)
Flexion	430.528 (*p* = 0.000)	1.944 (*p* = 0.174)	1.792 (*p* = 0.191)
Side Flexion Lt	100.885 (*p* = 0.000)	0.365 (*p* = 0.551)	0.718 (*p* = 0.404)
Side Flexion Rt	91.475 (*p* = 0.000)	0.451 (*p* = 0.508)	0.253 (*p* = 0.619)

VAS, Visual Analogue Scale; ODI, Oswestry Disability Index; Lt, Left; Rt, Right.

### 4.1. Pain (VAS Rest and Activity)

An analysis of variance was done to discover the effects of intervention between the groups that had shown an improvement in VAS values (rest and activity) after four weeks, which were statistically significant (Table 2). A *post hoc* Tukey’s HSD test showed that Group I showed significantly better improvement than group II after completion of the four weeks. Paired sample test, pre-post intervention showed as statically significance. 

### 4.2. Oswestry Disability Index (ODI)

An analysis of variance was done to discover the effects of intervention between the groups that had shown an improvement in the values of ODI after four weeks, which were statistically significant (Table 2). A *post hoc* Tukey’s HSD test showed that Group I showed significant improvement as opposed to Group II after completion of the four weeks.

**Table 3:** Paired Sample Test to observer the Pre-Post intervention effect.

Paired Samples Test									
		Paired Differences					t	df	Sig. (2-tailed)
		Mean	Std. Deviation	Std. Error Mean	95% Confidence Interval of the Difference				
					Lower	Upper			
Pair 1	RPRE–RPOS	5.167	1.117	0.204	4.750	5.584	25.341	29	0.000
Pair 2	APRE–APOST	5.700	1.088	0.199	5.294	6.106	28.707	29	0.000
Pair 3	OPRE–OPOST	43.200	9.404	1.717	39.688	46.712	25.160	29	0.000
Pair 4	EPRE–EPOST	−1.8333	0.5921	0.1081	−2.0544	−1.6122	−16.959	29	0.000
Pair 5	FPRE–FPOST	−2.067	0.553	0.101	−2.273	−1.860	−20.472	29	0.000
Pair 6	LPRE–LPST	−1.383	0.751	0.137	−1.664	−1.103	−10.093	29	0.000
Pair 7	RPRE–RPOST	−1.267	0.716	0.131	−1.534	−0.999	−9.690	29	0.000

PRE, VAS at rest (Pre); RPOS, VAS at rest (Post); APRE, VAS at activity (Pre); APOST, VAS at activity (Post); OPRE, ODI (Pre); OPOST, ODI (Post); EPRE, Extension (Pre); EPOST, Extension (Post); FPRE, Flexion (Pre); FPOST, Flexion (Post); LPRE, Side Flexion Left (Pre); LPST, Side Flexion Left (Post); RPRE, Side Flexion Right (Pre); RPOST, Side Flexion Right (Post).

### 4.3. ROM (Lumbar Extension, Flexion and Side Flexion)

An analysis of variance was done to discover the effect of intervention, which did not show any statistically significant differences between the groups in the values of all ROM after four weeks, but there was significant effect for the time in both the groups (Table 2). A *post hoc* Tukey’s HSD test did not show any statistically significant differences between the groups after the completion of the four weeks.

Reduction of pain in the Maitland group is probably due to the neuro-physiological and psychological effects [15] of mobilization. Pain gate theory [16] has shown that central transmission of pain can be blocked by increased pro prioceptive input. As nociceptor activity giving rise to pain generates reflex effects, in same way, PPIVM (Passive Physiological Inter-Vertebral Movements) also generates reflex effects [17]. According to Maitland, posterio-anterior pressure over the spinous process of vertebrae increases intervertebral physiological movements, this, in turn, increases proprioception. As a result, there is decreased pain perception.

As given by Maitland, a low amplitude graded oscillatory procedure has more of a tissue deformation (stretching) effect, whereas a single high velocity procedure has more of a pro prioceptive (neurological) effect. Three possible mechanisms as to which Maitland mobilization is effective in lumbar spondylosis are: (a) In the case of chronic low back pain, mechanoreceptors in the spinal stabilizing system are injured and nociceptors are activated, which generate corrupted transducer signals to the NM (Neuro-muscular) control unit, which, in response, generates corrupted muscle response patterns. Maitland mobilizations stimulate mechanoreceptors and inhibit nociceptors, which send normal signals to the NM control unit, which generates muscle response patterns to activate and coordinate spinal muscles to provide muscle mechanical stability. (b) Central postero-anterior mobilization stretches tightened capsules, muscles, ligaments, and fibrosed disc so it helps in regaining the mobility that was restricted due to these tight structures. (c) Patients with lumbar spondylosis have decreased lumbar lordosis, which signifies increased muscle activity leading to muscle fatigue when maintaining a posture. However, with central PA (Postero-Anterior) mobilization, the normal lordosis is restored so muscle activity is decreased and muscles are able to relax, thus decreasing the pain and increasing ROM. According to Triao, spinal mobilization uses controlled forces and moments applied to the spine, along with inertial forces generated by acceleration of relevant body segment masses. The algebraic sums of these loads are transmitted to the spine in a controlled manner and are designed to “unbuckle” motion segments and reduce local mechanical stresses within the functional spinal unit. As a limitation to this study, we feel that further studies are required, with small sample sizes, to see the generalizability of the study.

## 5. Conclusions

Our study has demonstrated that Maitland mobilization is more effective in managing low back pain and function of the lumbar spine than conventional treatments (traction and stretching exercises), but both groups are effective without a significant difference in improving the range of motion of the lumbar spine in patients with chronic low back pain due to lumbar spondylosis. Maitland mobilization in lumbar spine spondylosis should be given preference, as it helps to reduce pain and impaired function to a greater extent than conventional therapy. However, prospective studies are required to observe long term effects, with follow ups to establish the effect of Maitland mobilization in subjects with chronic low back pain due to lumbar spondylosis.
